# Plication versus Resection in Horizontal Strabismus Surgery: A Systematic Review with Meta-Analysis

**DOI:** 10.1155/2020/5625062

**Published:** 2020-07-02

**Authors:** Dayane Cristine Issaho, Denise de Freitas, Monica Fialho Cronemberger

**Affiliations:** ^1^Hospital de Olhos Do Parana, Curitiba, Brazil; ^2^Department of Ophthalmology and Visual Sciences, Federal University of Sao Paulo, Sao Paulo, Brazil; ^3^Post-Graduation Program (PhD) in Ophthalmology and Visual Sciences, Federal University of Sao Paulo, Sao Paulo, Brazil

## Abstract

**Purpose:**

The purpose of this review was to compare the efficacy of rectus muscle plication versus resection on the treatment of horizontal strabismus and to evaluate the exodrift after each technique.

**Methods:**

A research was performed in Latin American and Caribbean Literature on Health Sciences (LILACS); MEDLINE; and Cochrane Central Register of Controlled Trial (CENTRAL). The database was searched by 30 June 2019. The selection was restricted to articles published in English, Spanish, or Portuguese. There were no date restrictions in the search. A minimum mean follow-up of six months was required to access the primary outcomes. Motor alignment success was defined as postprocedure deviation within 10 prism diopters (PD) of orthotropia.

**Results:**

Seven studies were eligible for inclusion. The grouped success rate after plication was 66% (95% CI =  [43%–89%]), and the grouped success rate after resection was 68% (95% CI = [43%–89%]). High heterogeneity was observed between the estimations. There was no difference between the mean amount of deviation corrected in prism diopters, when using the mixed-model approach (SMD = 0.12; 95% CI = −0.2–0.44; *p*=0.45). The undercorrection rates were also analyzed. The combined odds ratio was 1.37 (95% CI = 0.59–3.16; *p*=0.462), and there was no statistical significance.

**Conclusion:**

Plication of horizontal extraocular muscles reveals to be an alternative to resection in strabismus surgery, with similar results. Exodrift is observed after plication and after resection in the treatment of exotropia, but randomized clinical trials are necessary to analyze and compare the follow-up.

## 1. Introduction

Resection is a traditional muscle-tightening procedure in strabismus surgery in which the muscle is disinserted from the globe and it is shortened. In contrast, plication is a tightening procedure in which the muscle is not dissected from its site of insertion [1–3].

The progress in ocular imaging, especially ocular coherence tomography angiography (OCTA), has shown how the iris vascular density on OCTA reduces after standard strabismus surgery [4]. Anterior segment ischemia is a rare but serious complication of strabismus surgery, and regarding this, plication is considered a less invasive technique, as the muscle is folded and sutured to the sclera at the insertion, preserving the anterior ciliary vessels and permitting simultaneous operations on multiple rectus muscles [3, 5, 6]. Several other advantages of plication have been described in the previous studies, including the absent risk of lost muscle, the possibility of early reversibility, and its relative simplicity and short operating time, as the prospect of less surgical trauma, inflammation, and hemorrhage than resection procedures [7–9].

The surgical effects of muscle-to-sclera plication to manage strabismus have been previously reported by several authors, and divergence has arisen as to whether the dose-response effect of horizontal rectus muscle plication corresponds to that of resection. Some authors advocate that plication has a similar effect per millimeter as resection to treat esotropia and constant exotropia [5, 10]. Others reported that muscle plication is less effective [11]. Also, as the amount of exodrift through time is not always predictable, many physicians plan the surgical doses intentionally to overcorrect in the early postoperative period.

### 1.1. What Is the Importance of This Review?

Currently, there is no consensus on performing muscle-to-sclera plication or resection in the treatment of horizontal strabismus. In the past decades, several retrospective case series have been published on plication to treat horizontal and vertical deviations. The studies were performed in different types of strabismus, methods, and follow-up times. The outcomes were diverse.

The main purposes of this study were to compare the efficacy of rectus muscle plication versus resection on the treatment of horizontal strabismus and to evaluate the average exodrift after surgery with each technique.

## 2. Methods

### 2.1. Search Methods for Identifying Studies

A research was performed in Latin American and Caribbean Literature on Health Sciences (LILACS), MEDLINE, and Cochrane Central Register of Controlled Trial (CENTRAL). The database was searched by 30 June 2019. The selection was restricted to articles published in English, Spanish, or Portuguese. We reduced the possibility of language bias by also analyzing all the abstracts published in English. There were no date restrictions in the search.

The strategies used were as follows: plication, tucking, resection, strabismus surgery, strabismus, esotropia, and exotropia. See Appendices for details of search strategies for CENTRAL (Appendix A), MEDLINE (Appendix B), and LILACS (Appendix C).

### 2.2. Study Selection

Two review authors independently screened the titles and abstracts obtained by the electronic searches and obtained full-text copies of definitely or potentially relevant studies. We contacted the corresponding investigators to obtain the missing data and allowed a time of three months for response. Nonresponse was reported as missing data. Any disagreements at any stage of screening were resolved by a third review author.

We included trials in which rectus muscle plication or resection was performed to treat horizontal strabismus and compared the efficacy of the treatment.

### 2.3. Data Collection and Risk of Bias Assessment

Two of the authors selected and extracted the data from the studies. We searched for the following elements:

- Methods: inclusion and exclusion criteria, follow-up period, and success criteria
- Participants: age at which the procedure was performed and the history of previous treatment
- Intervention: type of procedure performed
- Outcomes: motor outcomes after a minimum mean follow-up of 6 months
- Bias

A minimum mean follow-up of six months was required to access the primary outcomes. Motor alignment success was defined as postprocedure deviation within 10 PD of orthotropia and was considered the primary beneficial outcome.

The following analyses were performed:

- Success rate
- Undercorrection rate (remaining deviation >10 PD)
- The mean variation of the deviation after horizontal rectus muscle plication and after resection

We assessed the study quality according to the methods set out in Chapter 8 of the Cochrane Handbook for Systematic Reviews of Interventions [12]. We used the Cochrane tool for assessing risk of bias. We assessed sequence generation, allocation concealment, personnel and outcome assessors, incomplete outcome data, selective outcome reporting, and other sources of bias. We did not restrict the study design.

Masking of participants and physicians was not possible with the interventions considered for this review. However, it was possible to mask the assessment of treatment outcomes by having vision and orthoptic testing completed by different orthoptists. We did not exclude studies based on risk of bias assessments but conducted sensitivity analyses to assess the impact on the summary effect from studies with unclear or high risk of bias. We solved the discrepancies through discussion.

### 2.4. Eligibility Criteria

The criteria for considering studies for this review were as follows: studies analyzing neurologically normal patients with horizontal strabismus; minimum mean follow-up of 6 months; rectus muscle plication or resection performed during strabismus surgery; and criteria of success postoperative deviation within 10 PD in primary position.

### 2.5. Exclusion Criteria

The criteria for excluding studies for this review were as follows: prior strabismus surgery on the involved rectus muscles; oculomotor or abducens nerve palsy in the population studied; abnormal eye muscles operated (Graves' disease, Duane syndrome, and prior scleral buckle surgery); population studied including plication or resection performed in vertical rectus muscles; and plication using an adjustable suture technique.

### 2.6. Data Synthesis and Analysis

The comparison of the deviation changes before and after surgery was performed by standardized mean difference. The comparison of the success and undercorrection proportions were performed by odds ratio. Additionally, we presented the grouped success proportions for each type of procedure.

The heterogeneity between studies was evaluated by the use of Cochran's Q test and the *I*^2^ statistics [12]. The *I*^2^ statistics varies from 0 to 100, and the higher it is, the greater the heterogeneity observed among the rates [13].

All the meta-analyses were performed using random-effect models, due to the high heterogeneity between the studies' results.

As we have a small number of studies with unequal sizes, *t* statistic was used for adequate error rates [14].

A significance level of 5% was used for all statistical tests. Statistical analyses were performed using statistical software STATA 12.0 (Stata Corp., College Station, Tex.).

This manuscript was performed according to the PRISMA statement for systematic reviews and meta-analyses [15].

## 3. Results

The electronic searches identified a total of 58 titles and abstracts, and we requested the full text of 17 studies (Table 1).

We included a total of 7 studies for analysis.

### 3.1. Description of Studies

Details of the 7 included studies can be found in Tables 2 and 3.

Kimura and Kimura [16] compared plication-recession (PR) with resection-recession (RR) in 88 adult patients with intermittent exotropia. Forty-five patients were submitted to plication and 43 to resection. Patients were treated at a mean age of 42 and 34.7 years, respectively. The preoperative deviations were 40.1 ± 12.9 PD and 40 ± 14.9 PD, and the variations were 30.5 PD and 27.6 PD in the last visit (mean follow-up 21 and 24 months, respectively). 55% (45% undercorrected) and 50% (50% undercorrected) of the patients were within 10 PD of orthotropia in the last follow-up (*p* 0.66). The total exodrift from the last follow-up to 1 week after surgery was significantly less in the PR than in the RR group (1 month to 1 week, *p* 0.03; last follow-up to 1 week, *p* 0.04), but there was no difference between the two groups at other time points.

Wang et al. [17] in a prospective study analyzed 55 patients with convergence insufficiency-type intermittent exotropia. The authors divided the patients in two groups according to procedure (27 who were submitted to bilateral medial rectus plication and 28 to resection). The mean ages at surgery were 10.4 and 9.1, respectively. Mean preoperative deviation was 49.4 ± 13.5 PD and 52.4 ± 11.9 PD, and 6 months after surgery, it reduced to 19.4 ± 13.5 PD and 18.4 ± 12.9 PD. Success rates were 64% (36% undercorrected) and 62% (38% undercorrected); *p* 0.86. The greatest amounts of exodrift were observed during the first postoperative month in both groups. The total exodrift from 6 months to the first day after surgery was 19.6 PD and 21.6 PD.

Sukhija et al. [18] recruited patients with exotropia of 30 PD to 50 PD who had undergone first-time strabismus surgery and prospectively underwent UBM evaluation 1 year after surgery. Patients were divided into two groups according to whether the medial rectus was plicated (13 patients) or resected (15 patients), combined with antagonist lateral rectus recession. The mean initial deviation was 46.5 ± 4.7 PD and 43.2 ± 5.2 PD, and after a mean time of 14.26 months and 14.5 months, respectively, it reduced to a mean deviation of 3.33 PD in both groups (*p* 0.81). All the patients were within 10 PD of orthotropia in the last follow-up.

Alkharashi et al. [11] evaluated the results of 24 patients submitted to plication and 46 patients submitted to resection to treat strabismus. The vertical strabismus cases were excluded from our analysis. The mean preoperative deviation was 32 PD in the plication group and 30 PD in the resection group. After a mean follow-up of 11 months and 22 months, success rates were 58% and 89%, respectively (*p* 0.03). Exodrift was observed in both groups, with a mean variation of 15 PD and 11 PD (*p* 0.4).

In another research, Chaudhuri and Demer [5] evaluated 53 patients in whom plication or resection combined with antagonist rectus recession was performed to treat horizontal strabismus. For statistical analysis, we considered the groups separately (group A: exotropia; group B: esotropia). In the exotropia group, 9 patients had plication and 19 had resection. The mean medial rectus plication performed was 6.4 ± 1.4 mm, and the mean resection was 5.1 ± 1.1 mm. The mean preoperative deviations were 32.8 ± 14.4 PD and 31.2 ± 15.6 PD. Variation after surgery was 31.2 PD and 29 PD (mean follow-up of 4.87 and 32.2 months, respectively). The exotropia postoperative correction was 8.08 PD/mm for plication and 6.81 PD/mm for resection.

In group B (esotropia) [5], 13 patients had plication and 12 had resection. The mean lateral rectus plication performed was 6.5 ± 2.2 mm, and the mean resection was 6.6 ± 1.6 mm. The mean preoperative deviations were 27.9 ± 13.4 PD and 29 ± 15.2 PD. Variation after surgery was 26.1 PD and 27 PD (mean follow-up of 4.3 and 56 months, respectively). The esotropia postoperative correction was 5.17 PD/mm for plication and 6.63 PD/mm for resection.

Huston and Hoover [19] compared the results of rectus muscle plication versus resection combined with antagonist muscle recession for basic horizontal strabismus in 162 patients with esotropia and 60 patients with exotropia. For statistical analysis, here we also considered the groups separately (group A: esotropia; group B: exotropia). In the exotropia group, 31 patients had plication (mean age 34 years) and 29 had resection (mean age 23 years). The mean medial rectus plication was 5.4 ± 0.93 mm, and the mean resection was 4.1 ± 1.53 mm. The mean preoperative deviations were 35.1 ± 7.6 PD and 28.8 ± 8.9 PD. Variation after surgery was 30.6 PD and 26.3 PD (mean follow-up of 10.2 and 26.2 months, respectively). Success rates were 97% and 77%.

In group B (esotropia) [19], 88 patients had plication (mean age 23 years) and 74 had resection (mean age 10 years). The mean lateral rectus plication was 6.9 ± 1.1 mm, and the mean resection was 7 ± 1 mm. The mean preoperative deviations were 30.4 ± 6.1 PD and 29.97 ± 5.47 PD. Variation after surgery was 27.7 PD and 26.9 PD (mean follow-up of 10.9 and 38.4 months, respectively). Success rates were 96% and 89%.

Lastly, Lee and Kim [20] compared the long-term surgical outcomes between unilateral lateral rectus recession associated with medial plication (RP) or resection (RR) in children with intermittent exotropia. 72 patients submitted to plication (mean age 6.6 years) and 114 to resection (mean age 6.7 years) with a minimum follow-up period of 2 years were retrospectively reviewed. Before surgery, the mean deviation was 27.3 ± 15.5 PD and 29.2 ± 5.3 PD in the in the first group and second group, respectively. After a mean time of 27.2 months and 42.9 months, the mean deviations were 15.5 PD and 13.4 PD. Success rates were 26.4% and 42.1% (*p* 0.021). The exodrift within time was 22 PD and 21.9 PD.

### 3.2. Statistical Analysis

In all the analyses, we used random-effect models, due to the high heterogeneity between the results of the studies.

**Figure 1:**
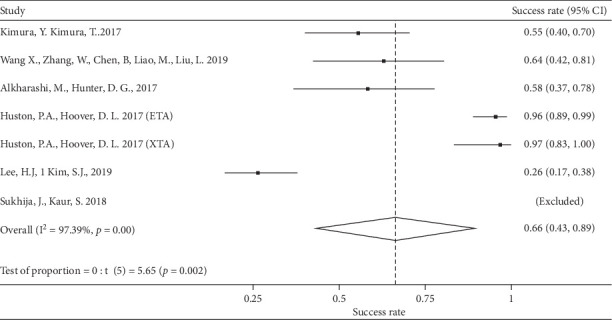
Grouped success rate-plication group.

The grouped success rate for plication was 66% (95% CI = [43%–89%]). Only the first three studies presented similar rates (between 50% and 87%) [16–18] (Figure 1).

**Figure 2:**
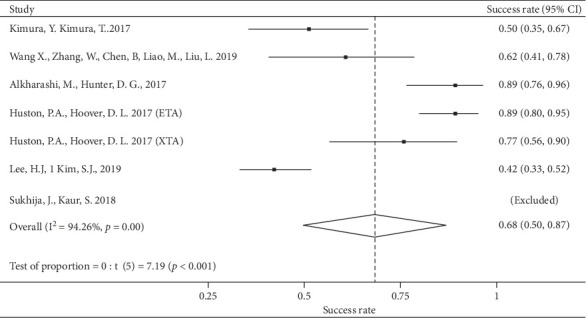
Grouped success rate-resection group.

The grouped success rate for resection was 68% (95% CI = [43%–89%]) (Figure 2).

**Figure 3:**
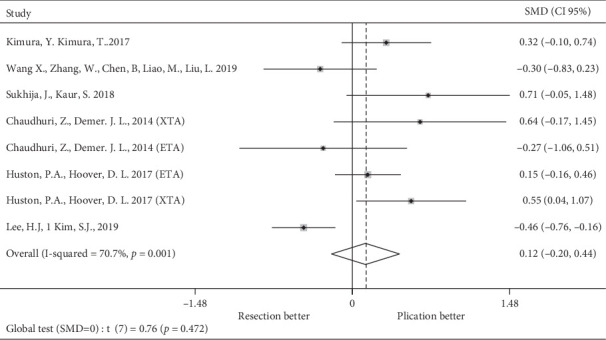
Standardized mean difference of deviation corrected.

As we notice in Figure 3, there was no difference between the mean amount of deviation corrected in prism diopters (SMD = 0.12; 95% CI = −0.2–0.44; *p*=0.45).

As shown in Table 4, the combined odds ratio for success was 0.97 (95% CI = 0.43–2.22; *p*=0.946), and there was no statistical significance.

When analyzing the undercorrection rates (Table 5), the combined odds ratio was 1.36 (95% CI = 0.59–3.16; *p*=0.462), and there was no statistical significance.

## 4. Discussion

Muscle-to-sclera plication is an alternative procedure to resection for tightening horizontal and vertical rectus extraocular muscles [1]. Plication has been first described in 1991 in an experimental study in monkeys [7]. Nevertheless, this muscle-tightening procedure has only recently received more attention in the literature. Possible barriers to a better acceptance of plication may be the uncertainty regarding its surgical efficacy and dose effect compared with that of the recognized resection.

Advantages of plication over resection include technical simplicity, potential reversibility, shorter operating time, less surgical trauma, preservation of anterior ciliary vessels, and absent risk of a lost or slipped muscle [5–7]. As plication is formed with absorbable sutures that, in case of Vicryl®, absorbs over a period of approximately 60 days, it is possible that the long-term effects might be inferior to resection.

In 2017, Wright described an innovative type of plication to correct diplopia associated with adult divergence insufficiency esotropia [21]. The lateral rectus central plication procedure tightens the slackened rectus muscles and minimizes the mild incomitance typical of these patients. Both the lateral rectus central plication and medial rectus recession were equally successful in eliminating diplopia, with over 90% success.

Here, we selected studies where plication and resection were compared. The underlying studies were of sufficient quality and similarity to warrant a meta-analysis.

Wright and Lanier reported that muscle plication was somewhat less powerful than a standard resection. They recommended increasing the posterior placement of the suture 0.5 mm compared with a standard resection [7]. Chaudhuri and Demer concluded that horizontal rectus muscle plication had an equal surgical effect to resection for the treatment of esotropia and exotropia [5]. Alkharashi and Hunter reported that 6–12 weeks postoperatively, surgical success was significantly higher in the resection group (89%) compared with that in the plication group (58%) [11].

It remains unclear how effective and predictable the muscle plication is in comparison with resection to treat strabismus.

Seven studies in which plication or resection was performed to treat horizontal strabismus were included in this analysis. For statistical analysis, we considered the groups separately (group A: exotropia; group B: esotropia) and presented the results independently. Only two reports were subdivided for analysis [5, 19]; therefore, a stratified examination considering different groups was not performed. Additional analysis revealed that when excluding the esotropia groups from statistics, the results were not affected. Hence, including the esotropia groups' results was not the reason for the high heterogeneity between the studies.

The heterogeneity between the studies' outcomes may be related to the different follow-up times considered by the authors. We attempted to reduce the bias by including in this review only studies with a minimum mean follow-up of 6 months, as we know that exodrift is expected in a recent postoperative time. Also, in all the statistical analyses, we used random-effect models, and the heterogeneity was evaluated by the use of Cochran's Q test and the *I*^2^ statistics [12].

We were able to report the primary outcome of similar improvement in ocular alignment by a comparable reduction in the mean deviation in all seven studies (combined standardized mean difference 0.12 [95% CI = −0.2–0.45]). Mean preoperative deviation for plication was 35.72 PD, and for resection, it was 34.87 PD. Mean postoperative deviation for plication was 7.3 PD, and for resection, it was 7.18 PD.

The grouped success rate of the plication group was 66%, and for resection, it was 68%, but we did observe a high heterogeneity in the studies (*I*^2^ statistics 97.4% and 94.3%, respectively).

Also, the combined odds ratio for success (Mantel–Haenszel) between plication and resection did not show statistical significance (0.97 [95% CI = 0.43–0.22]).

The chances for undercorrection were also comparable between groups ((Mantel–Haenszel) 1.36 [95% CI = 0.59–3.16]).

It was not possible to compare the amount of exodrift between the last follow-up and the immediate postoperative because pattern deviation was described in only one article [16]. But, in general, we did observe the numerical similarity on the amount of exodrift between groups in the postoperative follow-up.

The overcorrection rate was analyzed in four studies in which patients were treated for exotropia [11, 16–18]. Overcorrection was observed in 0.13% of those submitted to plication of medial rectus by one of the authors [11]. None of the other groups presented esotropia after plication or resection.

The study with the longer follow-up showed the worst success rates (26.4% in the plication group and 42.1% in the resection group) [20]. The difference between the success rates in a minimum follow-up of 2 years was statistically different between groups (*p*=0.021), although the differences on the final alignment were similar (*p*=0.97). Also, the amount of exodrift was similar between groups (22 PD and 21.9 PD). Interestingly, surgery was performed using a formula based on the surgeon's experience, and plication tended to be performed 1.5 mm more than resection for the same angle of deviation. The authors concluded that in children with intermittent exotropia, resection presented better surgical outcomes than plication. Additionally, initial overcorrection was important to achieve a good alignment.

Four authors attempted to analyze the binocular vision. Kimura and Kimura [16] demonstrated stereoacuity only before surgery, and there was no difference between groups (*p*=0.23). Wang et al. [17] showed that near stereopsis was improved in 44% patients in the plication group and 54% in the resection group (*p*=0.48). Chaudhuri and Demer [5] showed that stereopsis improved in 6 of 9 patients submitted to plication and 7 of 19 patients submitted to resection in the exotropia group. In the esotropia group, 5 of 13 patients improved stereopsis in the plication group and 2 of 12 patients improved in the resection group. Finally, Lee and Kim [20] showed similar binocular vision between groups before and after surgery.

When we perform a strabismus surgery, we always need to consider the risk of experiencing anterior segment ischemia in older patients and whenever more than 2 rectus muscles are operated in the same eye [4, 22, 23]. Sometimes, complications in a healthy eye are negligible, but this should still be cogitated. In addition to the procedural simplicity of plication, it allows simultaneous operations on multiple rectus muscles, can be considered a less invasive technique, and revealed to be a corresponding alternative to resection in strabismus surgery.

Our results elucidate many questions currently experienced on tightening procedures to treat horizontal strabismus. However, most of published literature consists of retrospective studies, case reviews, or cohort studies. There is not a standard method to describe plication; therefore, variability on results can lead to the fact that different surgeons are performing surgeries in different approaches. Moreover, different surgeons may operate with different anesthesiology practices (topical versus general), and this may have some impact in the outcome.

Although these provide very useful descriptive information, the outcome assessment was at a minimum mean of 6 months, and there was a range of postoperative timepoints. There is a strong necessity for good quality randomized trials to be performed in order to improve the evidence base for the use of plication as an option to shorten the extraocular muscle, since its effect corresponds to that of resection. Standardization is very important, considering the types and duration of strabismus. The evaluation of exodrift over a long follow-up time is also an important factor to be considered in the future trials.

## 5. Conclusion

Plication of horizontal extraocular muscles reveals to be an alternative to resection in strabismus surgery, with similar results.

Exodrift is observed after plication and after resection, but randomized clinical trials are necessary to analyze and compare the exodrift after surgery.

## Figures and Tables

**Table 1:** Results of the electronic search.

	MEDLINE	LILACS	CENTRAL
Plication AND resection AND strabismus	21	14	3
Plication AND resection AND strabismus OR esotropia OR exotropia	17	25	6
Tucking AND strabismus AND surgery	34	24	4

**Table 2:** Characteristics of included studies-group plication.

Study	Number of patients	Mean age at surgery (years)	Mean follow up (months)	Type of strabismus	Mean plication (mm)	Mean recession (mm)	Preop deviation PD	Variation on deviation PD	Success rate (%)	Undercorrection (%)	Exodrift PD
(1) Kimura and Kimura [16]	45	42	21	XT			40.1	30.5	55	45	6.8
(2) Wang et al. [17]	27	10.4	6	XT			49.4	30	64	36	19.6
(3) Sukhija and Kaur [18]	13	21.66	14.26	XT	6.19		46.5	43.17	100	0	
(4) Alkharashi and Hunter [11]	24	23	11	XT/ET			32		58	45	15
(5A) Chaudhuri and Demer [5]	9	38	4.87	XT	4.9	6.4	32.8	31.2			0.7
(5B) Chaudhuri and Demer [5]	13	38	4.3	ET	6.5	4.7	27.9	26.10			1.2
(6A) Huston and Hoover [19]	88	23	10.9	ET	6.91	4.6	30.37	27.65	96	3	
(6B) Huston and Hoover [19]	31	34	10.2	XT	5.4	7.03	35.07	30.59	97	3	
(7) Lee and Kim [20]	72	6.6	27.2	XT			27.3	11.8	26.4	73.6	22

**Table 3:** Characteristics of included studies-group resection.

Study	Number of patients	Mean age at surgery (years)	Mean follow up (months)	Type of strabismus	Mean resection (mm)	Mean recession (mm)	Preop deviation PD	Variation on deviation PD	Success rate (%)	Undercorrection (%)	Exodrift PD
(1) Kimura and Kimura [16]	43	34.7	24	XT			40	27.6	50	50	10.7
(2) Wang et al. [17]	28	9.1	6	XT			52.4	34	62	38	21.6
(3) Sukhija and Kaur [18]	15	24.86	14.5	XT	6.33		43.2	39.87	100	0	
(4) Alkharashi and Hunter [11]	46	15	22	XT/ET			30		89	0	11
(5A) Chaudhuri and Demer [5]	19	28	32	XT	5.1	6.7	31.2	29			1
(5B) Chaudhuri and Demer [5]	12	28	56	ET	6.6	5.2	29	27			1.5
(6A) Huston and Hoover [19]	74	10	38.4	ET	7.03	4.73	29.97	26.83	89	5	
(6B) Huston and Hoover [19]	29	23	26.2	XT	4.91	6.69	28.83	26.25	77	3	
(7) Lee and Kim [20]	114	6.7	42.9	XT			29.2	15.8	42.1	57.9	21.9

**Table 4:** Odds ratio for success.

Study	OR	95% CI	% weight
Kimura and Kimura [16]	1.19	0.52–2.76	
Wang et al. [17]	1.10	0.37–3.27	
Alkharashi and Hunter [11]	0.17	0.05–0.59	
Huston and Hoover [19]	2.55	0.74–8.82	
Huston and Hoover [19]	9.55	1.09–83.29	
Lee and Kim [20]	0.49	0.26–0.94	
Sukhija and Kaur [18]	(1)		
Combined OR (Mantel-Haenszel)	0.97	0.43–2.22	
Kimura and Kimura [16]	1.193	0.516–2.760	23.70
Wang et al. [17]	1.100	0.370–3.268	20.65
Alkharashi and Hunter [11]	0.171	0.050–0.586	18.96
Huston and Hoover [19]	9.545	1.094–83.294	10.67
Lee and Kim [20]	0.493	0.259–0.937	26.01
Sukhija and Kaur [18]	(Excluded)		
D + L pooled OR	0.805	0.334–1.941	100.00

OR (95% CI)–Odds ratio (95% Confidence interval). (1) It was not possible to estimate OR-100.0% had success in both groups. Heterogeneity test-Chi (5) = 17.84 *p*=0.003). *I*^2^ = 72%. Global test–*t*(5) = 0.07 (*p*=0.947). Heterogeneity chi-squared = 14.06 (d.f. = 4) *p*=0.007, *I*-squared (variation in OR attributable to heterogeneity) = 71.6%, Estimate of between-study variance Tau-squared = 0.6674, Test of OR = 1: *t*(4) = 0.48 Prob > |*t*| = 0.656.

**Table 5:** Odds ratio for undercorrection.

Study	OR	95% CI	% weight
Kimura and Kimura [16]	0.76	0.33–1.77	
Wang et al. [17]	0.91	0.31–2.70	
Alkharashi and Hunter [11]	79.22	4.38–1.433,38	
Huston and Hoover [19]	0.62	0.13–2.85	
Huston and Hoover [19]	0.93	0.06–15.65	
Lee and Kim [20]	2.03	1.07–3.86	
Sukhija and Kaur [18]	(1)		
Combined OR (Mantel-Haenszel)	1.36	0.59–3.16	
Kimura and Kimura [16]	0.764	0.330–1.766	27.96
Wang et al. [17]	0.909	0.306–2.700	24.35
Alkharashi and Hunter [11]	79.222	4.379–1433.382	8.32
Huston and Hoover [19]	0.933	0.056–15.647	8.67
Lee and Kim [20]	2.029	1.067–3.857	30.69
Sukhija and Kaur [18]	(Excluded)		
D + L pooled OR	1.610	0.620–3.857	100.00

OR (95% CI)–Odds ratio (95% Confidence interval). (1) It was not possible to estimate OR-100,0% had success in both groups. Heterogeneity test–Chi (5) = 13.20 (*p*=0.022). *I*^2^ = 62.1%. Global test–*t*(5) = 0.74 (*p*=0.493). Heterogeneity chi-squared = 12.13 (d.f. = 4) *p*=0.016, *I*-squared (variation in OR attributable to heterogeneity) = 67.0%, Estimate of between-study variance Tau-squared = 0.6647, Test of OR = 1: *t*(4) = 0.98 Prob > |t| = 0.382.

## Appendix

### A. CENTRAL Search Strategy

  #1 MeSH descriptor: [Esotropia] explode all trees
  #2 esotrop^∗^
  #3 convergen^∗^ strabism^∗^
  #4 #1 or #2 or #3
  #5 MeSH descriptor: [Exotropia] explode all trees
  #6 exotrop^∗^
  #7 divergen^∗^ strabism^∗^
  #8 #5 or #6 or #7
  #9 resection
  #10 plication
  #11 tucking

### B. MEDLINE Search Strategy

  1 esotrop
  2 exotrop
  3 strabism$ convergen$
  4 strabism$ divergen$
  5 resection
  6 plication
  7 tucking

### C. LILACS Search Strategy

  esotrop$ or converge$ or internal and strabism$
  esotropia
  exotropia
  exotrop$ or diverge$ or external and strabism$
  muscle resection
  muscle plication
  tucking
